# Endocrine disrupting chemicals: gestational diabetes and beyond

**DOI:** 10.1186/s13098-024-01317-9

**Published:** 2024-04-26

**Authors:** Tridip Mitra, Richa Gulati, Krithika Ramachandran, Rohan Rajiv, Elizabeth Ann L. Enninga, Chris K Pierret, Sajeetha Kumari R, Rajiv Janardhanan

**Affiliations:** 1 Division of Medical Research, Faculty of Medicine and Health Sciences, SRM Institute of Science and Technology, 603 203 Kattankulathur, Tamil Nadu India; 2 Dietrich School of Arts and Sciences, University of Pittsburgh, 15260 Pittsburgh, PA USA; 3 Department of Obstetrics and Gynecology, Mayo Clinic, Rochester, MN USA; 4 Department of Biochemistry and Molecular Biology, Mayo Clinic, Rochester, MN USA; 5 Department of Obstetrics and Gynecology, Faculty of Medicine and Health Sciences, SRM Institute of Science and Technology, 603 203 Kattankulathur, Tamil Nadu India

**Keywords:** Gestational diabetes mellitus (GDM), Endocrine-disrupting chemicals (EDCs), Transgenerational impact, Epigenetic regulation, Exosomal miRNAs

## Abstract

Gestational Diabetes Mellitus (GDM) has been on the rise for the last two decades along with the growing incidence of obesity. The ubiquitous use of Endocrine-Disrupting Chemicals (EDCs) worldwide has been associated with this increase in GDM incidence. Epigenetic modifications such as DNA methylation, histone acetylation, and methylation have been associated with prenatal exposure to EDCs. EDC exposure can also drive a sustained disruption of the hypothalamus-pituitary-thyroid axis and various other signaling pathways such as thyroid signaling, PPARγ signaling, PI3K-AKT signaling. This disruption leads to impaired glucose metabolism, insulin resistance as well as β-cell dysfunction, which culminate into GDM. Persistent EDC exposure in pregnant women also increases adipogenesis, which results in gestational weight gain. Importantly, pregnant mothers transfer these EDCs to the fetus via the placenta, thus leading to other pregnancy-associated complications such as intrauterine growth restriction (IUGR), and large for gestational age neonates. Furthermore, this early EDC exposure of the fetus increases the susceptibility of the infant to metabolic diseases in early life. The transgenerational impact of EDCs is also associated with higher vascular tone, cognitive aberrations, and enhanced susceptibility to lifestyle disorders including reproductive health anomalies. The review focuses on the impact of environmental toxins in inducing epigenetic alterations and increasing the susceptibility to metabolic diseases during pregnancy needs to be extensively studied such that interventions can be developed to break this vicious cycle. Furthermore, the use of EDC-associated ExomiRs from the serum of patients can help in the early diagnosis of GDM, thereby leading to triaging of patients based on increasing risk factor of the clinicopathological condition.

## Introduction

Gestational Diabetes Mellitus (GDM) is known to be the most common pregnancy complication among women worldwide and is usually diagnosed in the second or third trimester of pregnancy. The increasing prevalence of GDM has been associated with an increase in maternal obesity and depending on the diagnostic criteria used, it is known to affect 6-25% of pregnant women [1]. The standardized prevalence rate of GDM in 2021 across the world was 14% based on the International Diabetes Federation Diabetes Atlas, 2021 [2]. Advanced maternal age, ethnicity, high-carb diets, pre-pregnancy obesity, and a family history of type 2 diabetes mellitus (T2DM) have all been identified as risk factors for GDM [3, 4]. Interestingly, more than 50% of GDM patients lack these conventional traits, suggesting the important role of these environmental variables [5]. However, the precise causes of GDM still remain unknown.

The increasing use of Endocrine Disrupting Chemicals (EDCs) also correlates with the rise in the incidence of GDM. EDCs are specialized exogenous compounds that have the potential to significantly alter normal endocrine signals [6]. They share structural similarities with some endogenous hormones and thus, can cause hormonal disruptions which result in obesity and related lifestyle disorders like cardiovascular disorders, T2DM, as well as developmental and reproductive disorders [6]. EDCs are widely used in various consumer products like food packaging, cosmetics, fabrics, water, detergents, and various industrial consumables. Humans have been exposed to these EDCs for many years via diet, agricultural pesticides, and various other daily consumables. Probable risks to public and human health have been greatly exacerbated by the widespread usage of EDCs and their association with chronic disorders [7].

Several EDCs like Bisphenol A (BPA), phthalates, Per- and Poly-fluoroalkyl Substances (PFAS), heavy metals, dioxins, parabens, and others have been associated with disorders such as impaired glucose metabolism, T2DM, reproductive disorders, cancer, metabolic disorders, cognitive disorders, and hypertension8–11. A growing body of research indicates that exposure to EDCs is especially dangerous during pregnancy because it can affect the fetus, thereby resulting in long-term developmental complications such as intrauterine growth restriction (IUGR) and preeclampsia12. Particularly, many studies suggest that exposure to EDCs is directly associated with the development of GDM among pregnant women13,14.

Pregnancy is accompanied by a minimum amount of insulin resistance in the body of the mother which is essential for the development of the fetus. This tightly regulated pathway is disrupted by the EDCs, which act as xenobiotic substances affecting the hypothalamus-pituitary-thyroid axis aiding an increased insulin resistance in the body of the mother leading to GDM [13, 14]. This results in hyperglycemic conditions in the maternal body. Placental exposure to EDCs promotes an alteration of placental functioning [8] including the disruption in the expression of placental miRs which is represented by an altered level of Exosomal micro RNAs (ExomiRs) in the maternal circulatory system representing placental health [15]. This leads to severe maternal and fetal outcomes among patients with GDM [15].

**Fig. 1:**
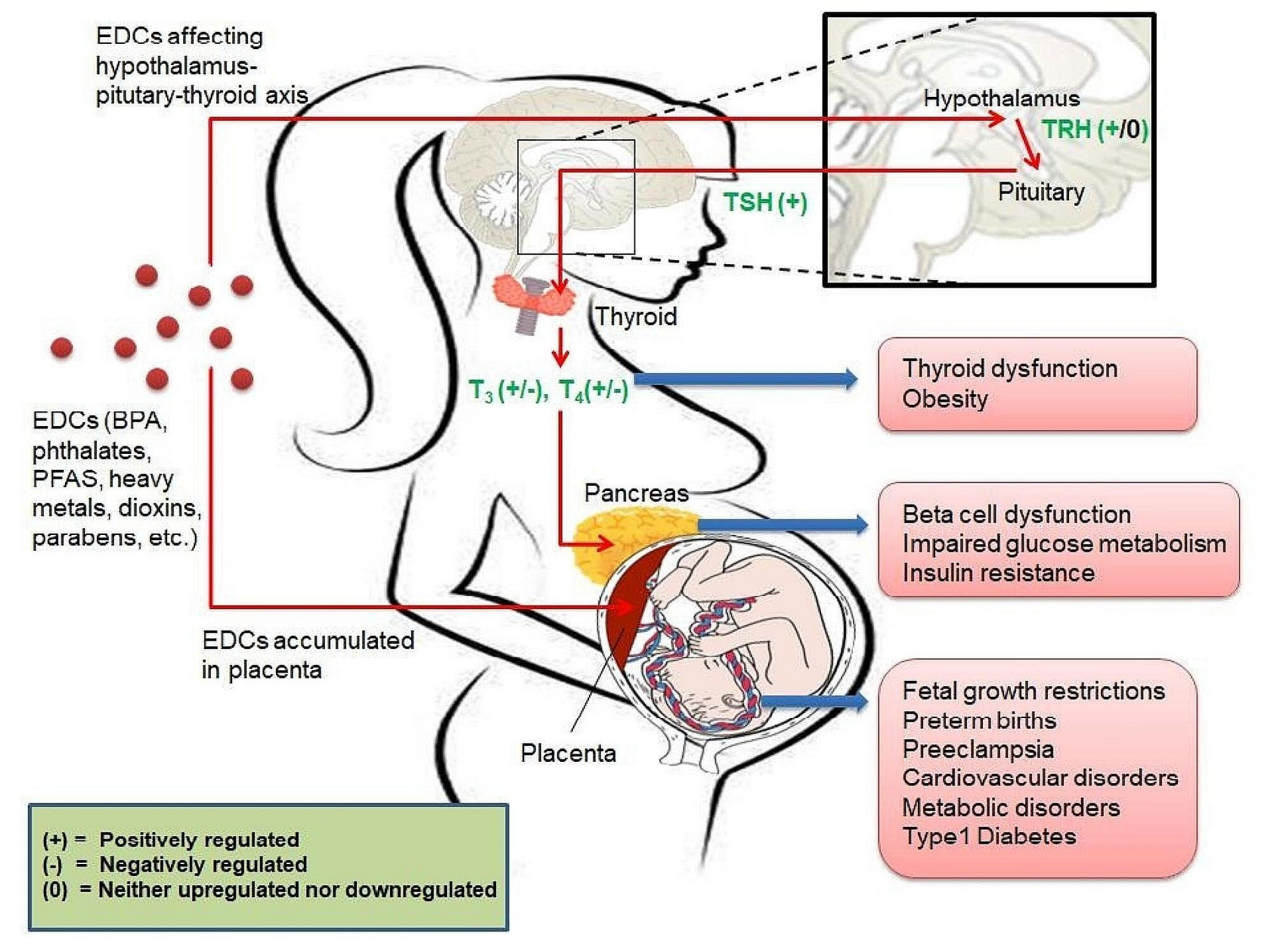
The various EDCs and their impact on pregnant women. EDCs can affect the hypothalamus-pituitary-thyroid axis and cause thyroid dysfunction, which in turn results in beta-cell dysfunction, impaired glucose metabolism, and insulin resistance among pregnant women. When the mother is exposed to EDCs, she can transfer them to the fetus via the placenta, which is one of the contributing factors for fetal growth restriction and preterm birth, as well as later development of cardiovascular and other metabolic disorders in exposed offspring. [EDC = Endocrine-Disrupting Chemical; BPA = Bisphenol A; PFAS = Per- and Poly-fluoroalkyl Substances; TSH = Thyroid stimulating hormone; TRH = Thyroid reducing hormone]

A mother can transfer EDCs to the developing fetus via the placenta [8]. This early fetal exposure to EDCs can lead to preterm birth as well as Type 1 Diabetes, obesity, and cardiovascular disease, and reproductive disorders in the offspring [9–11] (Fig. 1). Exposure of pregnant women to EDCs can also lead to various epigenetic modifications such as histone modifications and DNA methylation, which are associated with GDM onset among pregnant women and can hinder fetal development [12]. EDCs particularly disrupt the Hypothalamus-pituitary-thyroid axis, leading to β-cell dysfunction and impaired glucose metabolism [13] (Fig. 1). ExomiRs are promising candidates to act as non-invasive biomarkers and have been studied extensively in the context of several diseases [14]. However, there is a dearth of studies associating EDC exposure with epigenetic modifications, including alterations in ExomiRs in GDM patients. Exposure to EDCs has been hypothesized to alter exosomal signaling, which is a crucial mechanism for intercellular communication, and can contribute to GDM development as these circulating Exo-miRs are a surrogate marker of placental health in GDM, thereby acting as a placental function marker [15]. This review compiles the literature on mechanisms by which exposure to different EDCs results in GDM and related fetal developmental anomalies. It also discusses the transgenerational effect resulting from EDC exposure on children, who are more likely to have metabolic abnormalities later in life. We also highlight the role of epigenetic modifications, particularly the role of ExomiRs, in EDC-induced GDM development, which is an area in need of future research to develop non-invasive biomarkers as well as potential therapeutic targets for the prevention of GDM and its consequences. Furthermore, the establishment and utilization of a federated learning-based system for detecting GDM by utilizing m-health platforms will enable the most accurate detection of GDM and provide information on the software-based distribution of clinical resources as well as the detection of probable GDM-associated EDCs even in remote areas [15]. This would in turn help in the lifestyle interventions to reduce the dietary, behavioral, and residential exposure of EDCs to men and women of reproductive age [16] are seen to greatly influence the obstetrics health of women thereby improving pregnancy outcomes.

Gestation is a critical phase in the life of the mother and the fetus accompanied by suppressed immune functioning, any alterations during the phase can be the most probable cause for increased susceptibility in the offspring and the development of several complications in the offspring. This is often linked with the early onset of insulin resistance in the life of the offspring of mothers with GDM [9].. Moreover, the offspring of these mothers are also seen to have a higher basal metabolic index leading to obesity [9]. These infants are also seen to develop several anomalies in their early stages of life which include cardiovascular, respiratory as well as reproductive disorders [9–11]. This implies the Developmental Origins of Health and Disease (DOHaD) in offspring and the importance of the management of GDM.

## Literature search process

This review was performed by investigating key research and review papers presented at conferences and seminars, as well as publications in books, journals, and various other online resources. The publications for the literature study were mainly obtained from Google Scholar, PubMed, Science Direct, Springer, and Research Gate. This work includes results that were published over the previous 15 years. The most common keywords used to find publications were “Endocrine Disruptors,” “Gestational Diabetes,” and “microRNAs.” The term “AND” was additionally employed when searching for two or more keywords at the same time, to ensure no relevant publications were overlooked. The publications were manually reviewed and then filtered on the basis of the title, abstract, and content. The type and concentrations of EDCs mentioned in the study are depicted in tabular format (Table 1).

**Table 1:** List of association of various endocrine disruptors with GDM and other pregnancy outcomes

Sl. No.	EDCs Present and their Concentrations	Pregnancy complications	Study subjects	Samples collected	References
	Bisphenols				
1.	BPA (0.72 μg/L), BPF (1.74 μg/L), BPS (0.30 μg/L), BPAF (0.025 μg/L)	GDM	Human	Urine samples	20
2.	BPA (first trimester - 1.39 µg/L and second trimester - 1.27 µg/L	GDM	Human	Urine samples	22
3.	BPA (first trimester - 1.23 μg/L and second trimester - 1.01 μg/L)	GDM	Human	Urine samples	23
4.	BPA (2.05 µg/g)	GDM, Obesity	Mice	Urine samples	32
5.	BPA (0.34 ng/mL)	Pre-term births, congenital infection	Humans	Cord blood samples	33
6.	BPA (6.41 ng/mL)	GDM	Human	Urine samples	37
	**Phthalates**				
7.	MiBP (0.23 ng/mL), MBP (1.08 ng/mL), MBzP (0.15 ng/mL), MEHP (22.2 ng/mL)	GDM	Human	Urine samples	37
8.	MEP (0.07 ng/mL), MBzP (0.21 ng/mL), MCPP (0.33 ng/mL)	GWG, GDM	Human, Rats, Mice, Sheep, Baboons	Urine samples	44
9.	MEHP (1.2 ng/mL), MEP (0.5 ng/mL)	HDP, Obesity	Rats	Urine samples	50
	**Per- and Polyfluoroalkyl Substances (PFAS)**				
10.	PFAS (0.02–0.06 ng/mL)	GDM, Obesity	Mice	Blood samples	61
11.	PFOS (0.20 ng/mL), PFOA (0.20 ng/mL), PFHxS (0.20 ng/mL), PFNA (0.10 ng/mL)	GDM	Rats	Blood samples	62
12.	PFHxS (0.30 ng/mL), PFOS (8.31 ng/mL), PFOA (1.71 ng/mL), PFNA (0.66 ng/mL), PFDA (0.26 ng/mL)	GDM	Mice	Blood samples	63
13.	PFOS (0.20 ng/mL), PFAS (0.10 ng/mL), PFOA (0.30 ng/mL)	GWG	Human	Blood samples	65
14.	PFOS (27.2 ng/mL), PFOA (3.31 ng/mL), PFHxS (4.54 ng/mL), PFDA (0.28 ng/mL), PFNA (0.59 ng/mL)	GDM	Mice	Serum and cord blood samples	123
	**Heavy Metals**				
15.	Cr (2.286 μg/L), Mn (2.725 μg/L), Cu (932.164 μg/L), Zn (634.382 μg/L), Cd (0.096 μg/L)	GDM	Human	Blood samples	81
16.	Cd (0.37 μg/L), Pb (1.32 μg/L), Hg (0.93 μg/L)	HDP, Obesity, Insulin resistance	Human	Blood samples	82
17.	As (2.09–24.07 μg/L)	GDM	Human	Blood samples	84
18.	As (66 μg/L)	Teratogenic effects	Human	Urine samples	94
19.	Hg (11.9 μg/L)	GDM	Mice	Serum and cord blood samples	123
	**Parabens**				
20.	MeP (5.13 ng/mL), EtP (0.12 ng/mL), PrP (0.46 ng/mL)	GDM, Obesity	Mice	Urine samples	111
21.	MeP (17.96 μg/L), EtP (0.66 μg/L), PrP (0.94 μg/L)	GDM	Mice	Urine samples	112
22.	MeP (89.8 µg/L), PrP (19 µg/L), BuP (0.8 µg/L)	GDM	Human, Mice, Rats	Urine samples	113
	**Dioxins**				
23.	PCB (0.005 ng/mL)	GDM, Obesity	Mice	Blood samples	61
24.	PCB (1.23 μg/g-lipid)	GDM	Mice	Serum and cord blood samples	123
	**Other Endocrine Disruptors**				
25.	DDE (0.5 μg/g-lipid)	GDM	Mice	Serum and cord blood samples	123
25.	Nonylphenols (first trimester 4.10 ng/mL, second trimester 3.31 ng/mL, and third trimester 3.09 ng/mL)	GDM, Placenta previa, Preeclampsia, oligohydramnios Hyperthyroidism	Human	Urine samples	132

Abbreviations used in the table: GDM: Gestational Diabetes Mellitus; GWG: Gestational Weight Gain; HDP: Hypertensive Disorders of Pregnancy; BPA: Bisphenol A; BPF: Bisphenol F; BPS: Bisphenol S; BPAF: Bisphenol AF; MiBP: Mono-isobutyl phthalate; MBP: Monobutyl phthalate; MBzP: Monobenzyl phthalate; MEHP: Mono-2-ethylhexyl phthalate; MEP: Monoethyl phthalate; MCPP: Mono(3-carboxypropyl) phthalate; PCB: Polychlorinated biphenyls; PFAS: Per- and polyfluoroalkyl substances; PFOS: Perfluorooctane sulfonic acid; PFOA: Perfluorooctanoic Acid; PFHxS: Perfluorohexanesulfonic acid; PFNA: Perfluorononanoic Acid; PFDA: Perfluorodecanoic acid; Cr: Chromium; Mn: Manganese; Cu: Copper; Zn: Zinc; Cd: Cadmium; Pb: Lead; Hg: Mercury; As: Arsenic; MeP: Methyl Paraben; EtP; Ethyl Paraben; PrP: Propyl Paraben; BuP: Butyl Paraben; DDE: Dichlorodiphenyldichloroethylene

## Endocrine disrupting chemicals (EDCs) as a contributing factor to gestational diabetes mellitus (GDM)

### Bisphenol A (BPA)

The EDC with the biggest volume of production worldwide is BPA, with an emission rate of about 100 tons annually. Being the primary monomer in polycarbonate plastics, it is widely used and can enter the body orally and topically [17]. Various pieces of evidence suggest that BPA may be hazardous to human health, particularly for endocrine metabolism, including, but not limited to, glucose homeostasis [18–20]. Numerous epidemiological studies have been conducted across the world to find a link between the exposure of pregnant women to BPA and GDM. Some of these are described below, and these studies have found that the rise in GDM cases can be correlated with BPA exposure [21–23].

**Fig. 2:**
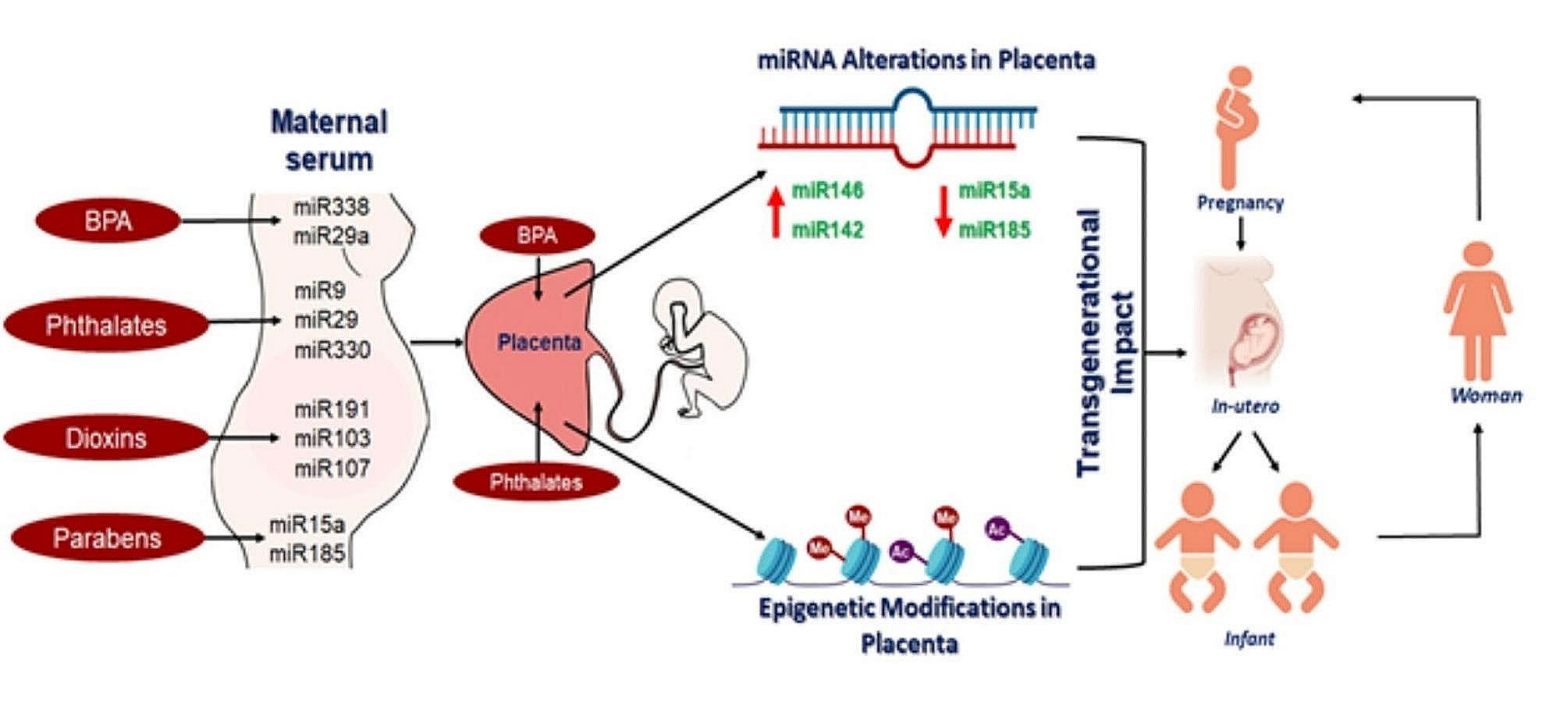
Exposure to EDCs like BPA, dioxins, phthalates and parabens alter certain microRNAs in maternal serum. BPA and phthalates alter placental microRNAs and induce epigenetic modifications such as histone methylation, histone acetylation, and DNA methylation, thereby having a transgenerational impact. There is a paucity of such epigenetic studies for EDCs like PFAS and heavy metals. [EDC = Endocrine-Disrupting Chemical; BPA = Bisphenol A; PFAS = Per- and Poly-fluoroalkyl Substances]

**Fig. 3:**
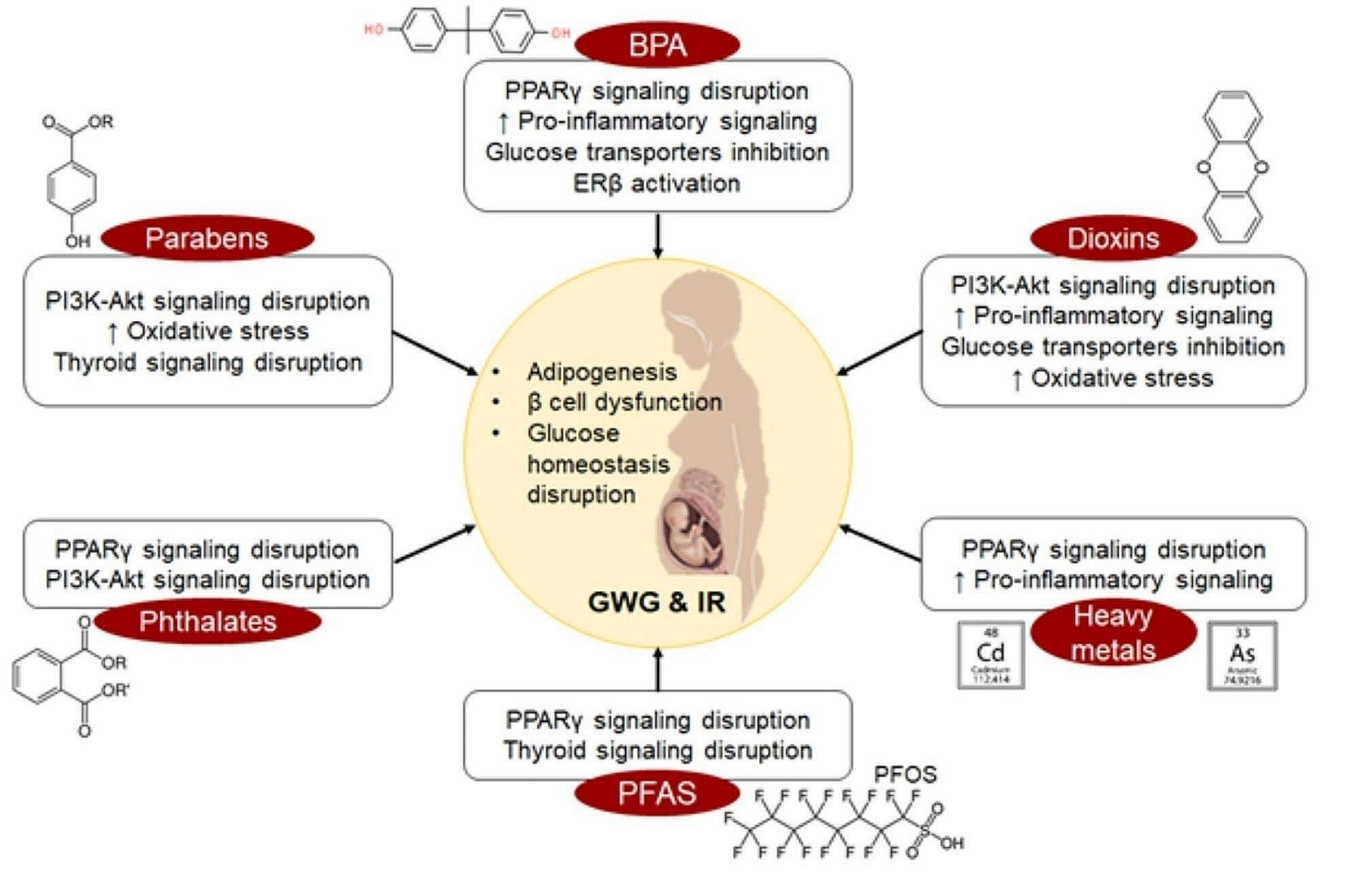
Exposure to EDCs like BPA, phthalates, PFAS, dioxins, heavy metals, and parabens among pregnant women disrupts various signaling pathways and leads to increased incidence of gestational weight gain and insulin resistance. Pi3K-Akt expression pathway is the most common pathway which is seen to be disrupted by the EDCs, leading to GDM. [EDC = Endocrine-Disrupting Chemical; BPA = Bisphenol A; PFAS = Per- and Poly-fluoroalkyl Substances; PPAR = Peroxisome proliferator-activated receptor; ER = Estrogen receptor; GWG = Gestational weight gain; IR = Insulin resistance]

According to one study, blood glucose levels in sub-fertile women were positively correlated with BPA exposure during the second trimester of pregnancy [23]. Additionally, among overweight/obese women, second-trimester urinary BPA concentrations are directly linked to their glucose levels [24]. Disruption of maternal glucose homeostasis by BPA is firmly supported by in vivo research, and this effect is mediated via estrogen receptor-β activation [25]. Estrogens have a significant role in increasing β-cell mass during rodent pregnancy in part due to miR-338-3p suppression [26]. A rodent study by Wei et al. demonstrates that B.P.A. exposure to mice leads to a lower expression of serum miR-338 which consequently suppresses the translation of the protein PDH1 expression in pancreatic tissues, resulting in an increasing irregularity of insulin secretion [27] (Fig. 2). Other in vitro studies have shown that BPA exposure may contribute to weight gain and the development of obesity through several pathways, including the proliferation and differentiation of 3T3L1 preadipocytes [28, 29]. Exposure to BPA is also seen to enhance the expression of IL6 in 3T3L1 cells [30]. Another study in human adipose stem cells showed that activation of estrogen receptors by BPA leads to the induction of adipogenic genes (DLK, PPARγ, IGF1, etc.) [31]. Angelo et al. observed that mice exposed to BPA showed a reduction in adiponectin secretion, thereby leading to obesity [32]. The link between BPA exposure and obesity has also been noted in humans. According to the NHANES survey, urine BPA levels are linked to obesity, which may indirectly contribute to the development of GDM [33]. Furthermore, an increase in prenatal BPA levels leads to the upregulation of pro-inflammatory cytokines such as TNFα and IL6 in neonatal cord blood giving rise to insulin resistance and β-cell dysfunction [34]. (Fig. 3). It is also been demonstrated that BPA in pregestational and gestational diabetes patients could decrease placental expression of glucose transporters (GLUT1, GLUT4, and GLUT9), whose levels are inversely correlated with maternal body mass index (BMI) during the course of gestation [35] (Table 2). Furthermore, exposure to EDCs like BPA is associated with an altered fibroglandular volume thereby altering the density of breast tissues [36]. These scattered fibroglandular breast tissue can often form lumps making women more susceptible to acquiring breast cancer even independent of the BMI [37].

Pregnant women can transfer BPA to the developing fetus via the placenta [38] allowing, exposure to BPA to have a transgenerational impact (Fig. 2). Exposure to BPA can lead to epigenetic modifications like miRNA alterations, increased DNA methylation, and altered histone methylation and acetylation patterns. For instance, a whole genome study compared the level of expression of 1349 miRNAs in placental samples from women who lived in areas that were polluted to those from women who lived in unpolluted areas using miRNA microarray technology. In this study, they found that significantly higher miR-146a expression, which targets genes associated with neural disorders, signal transduction, and cancer, was linked to high placental BPA levels [39] in the samples from polluted areas (Fig. 2). Other miRNAs including miR-29a, miR-222, and miR-132 have also been shown to be increased in GDM and this increase is attributed to BPA exposure [40, 41], further indicating the utility of using serum miRNAs as non-invasive biomarkers. Other than miRNA alterations, BPA exposure during rat pregnancy has also been shown to induce DNA hypermethylation of the *Igf2* gene in islets of the offspring, leading to its overexpression [42]. Similarly, another rat model showed that maternal exposure to BPA caused demethylation of H3K4 and H3K9 at the *Pdx1* promoter resulting in a downregulation of pancreatic *Pdx1* expression in the offspring [43]. Such epigenetic modifications can cause cardiovascular disorders, diabetes, childhood obesity, and other metabolic disorders among exposed infants [12, 44] (Table 2). Additionally, lifestyle modifications can lead to minimizing the risk of exposure to different bisphenols thereby reducing dietary, behavioral as well as residential exposure thereby improving the reproductive health of both men and women [16]. Furthermore, successful interventions in targeting the route for known exposures of Bisphenols to provide personalized education and support to participants thereby helping them tp replace the items known the be sources of Bisphenols have been taken by several governing bodies to improve the health of people [16].

### Phthalates

Since the 1930s, phthalates, also known as phthalic acid diesters, have been a pervasive class of synthetic compounds used as plasticizers in a variety of consumer products ranging from plastic bottles to cosmetics [45]. Phthalates do not have covalent bonds with polymers, therefore they can easily leach into the environment and enter the human body through ingestion, inhalation, or cutaneous absorption. Several studies highlighted below have described the endocrine-disrupting nature of phthalates, especially the primary priority phthalates, which can lead to various metabolic and reproductive disorders among women.

A study in pregnant women demonstrated that increased gestational weight gain (GWG) and decreased glucose tolerance are linked to higher urinary mono-ethyl phthalate (MEP), a metabolite of the parent molecule of di-ethyl phthalate (DEP) [46]. A positive association between urinary mono-(3-carboxypropyl) phthalate (MCPP) and GWG in pregnant women has also been described suggesting that phthalates can act as a precursor to GDM [47]. Mechanistically, phthalates have been shown to be PPARγ agonists (Fig. 3), and activation of PPARγ by phthalates stimulates the proliferation of adipocytes in 3T3L1 cells [48] as well as in primary mouse bone marrow cells [49]. An in vivo rat study showed that gestational exposure to the priority phthalate di(2-ethylhexyl) phthalate (DEHP) impaired glucose tolerance in offspring at 2 months of age [50]. A study by Chen et al. showed that in GDM-positive rats, di-n-butyl phthalate (DBP) increased hyperglycemia among pregnant women and impaired glucose handling in vivo, while it impaired FOXM1, decreased β-cell viability, and impaired STAT1 signaling cascade in vitro [51]. Another study in mice found that DBP exposure significantly increased glucose intolerance and insulin resistance by disrupting PI3K expression, AKT phosphorylation and decreased pancreatic GLUT2 expression [52] (Fig. 3). According to a prospective study, phthalate exposure in a low-risk cohort of pregnant women was associated with elevated diastolic blood pressure within the first 20 weeks of pregnancy as well as the emergence of Hypertensive Disorders of Pregnancy (HDPs) like gestational hypertension and pre-eclampsia in the latter stages of pregnancy [53]. In a study by Binder et al., it was shown that exposure to phthalates like DEP and MEP can also cause an increase in the breast fibroglandular volume thereby making women more susceptible to breast cancer [36].

Patients with GDM have altered levels of certain circulating miRNAs or ExomiRs, including miR-29a, miR-222, miR-132 [41], or miR-16, miR-17, miR-19, and miR-20a [54]. Another human study found a correlation between serum phthalates and miR29a expression. Specifically, a positive correlation was observed between mono-2-ethylhexyl phthalate (MEHP) and miR-29a, wherein both monobutyl phthalate (MBP) and mono-isobutyl phthalate (MiBP) levels were negatively correlated with miR-29a levels [40]. An elevated levels of serum miR-29a is seen in GDM patients compared to serum of non-diabetic pregnant women [40]. Apart from circulating miRNAs, phthalates have also been shown to alter the expression of various placental miRNAs such as miR-185, miR-142-3p, and miR-15a-5p, thereby leading to GDM among pregnant women [55] (Fig. 2). Other epigenetic modifications are also associated with phthalate exposure as has been noted in animal models. Rats exposed to di(2-ethylhexyl) phthalate (DEHP) showed an increase in global DNA methylation [50]. A study in mice showed that gestational phthalate exposure resulted in DNA methylation alterations in the sperm of F3 offspring and this increased the transgenerational inheritance of obesity as well as reproductive disorders [56]. Thus, phthalate exposure contributes to maternal obesity as well as GWG, and epigenetic modifications underlie these phenotypes [57]. This can eventually lead to insulin resistance and β-cell dysfunction which has a transgenerational impact. Later in life, offspring from mothers exposed to phthalates have an additional risk of developing obesity, diabetes, cardiovascular, and reproductive disorders [58] (Table 2). Furthermore, a study by Martin et al., shows that lifestyle modification changes greatly impact the exposure of men and women of reproductive age to phthalates thereby improving their reproductive health [16]. Such types of lifestyle modifications include prevention of dietary exposure (plastic water bottles, plastic kitchenware, canned food), behavioral exposure (personal care products, cosmetics, dental care), and residential exposure (PVC pipes, pipes) by providing personalized education to the people in the society, thereby spreading awareness among participants in replacement of the product which are considered as the source of phthalates [16].

**Table 2:** The table shows epigenetic alterations caused by various endocrine disruptors leading to GDM

Sl. No.	EDCs Present	Epigenetic alterations	References
1.	**Bisphenols** BPA, BPF, BPS, BPAF	miR level alterations (miR-146a, miR-29a, miR-222, miR-132), DNA hypermethylation, protein demethylation.	20, 22, 23, 32, 33,37
2.	**Phthalates** MiBP, MBP, MBzP, MEHP, MEP, MCPP	miR level alterations (miR-29a, miR-222, miR-132, or miR-16, miR-17, miR-19, and miR-20a, DNA methylation, DNA methyl alterations.	37, 44, 50
3.	**Per- and Polyfluoroalkyl Substances** PFAS, PFOS, PFHxS, PFNA, PFDA	miRs level alterations, DNA methylation changes.	61, 62, 63, 65, 123
4.	**Heavy Metals** Cr, Mn, Cu, Zn, Cd, Pb, Hg, As	Altered miR-191 levels, DNA methylation alterations.	81, 82, 84, 94, 123
5.	**Parabens** MeP, EtP, PrP, BuP	miR level alterations (miR-15a-5p, miR-185, DNA methylation.	111, 112, 113
6.	**Dioxins** PCB	miR level alterations (miR-191, miR-103, miR-107), DNA methylation.	61, 123

### Per- and polyfluoroalkyl substances (PFAS)

PFAS refers to a broad class of synthetic chemicals utilized in consumer and industrial products which includes shampoo, cosmetics, fast food packaging, non-stick cooktops, and pesticides [59]. They are resistant to degradation due to the robust carbon-fluorine bonds in their structure [59]. According to the Stockholm Convention, the use of PFAS has been phased out in many countries, however, because they can remain and accumulate in the environment, they continue to circulate even after their production has been stopped [60].

Rodent studies demonstrate that high prenatal exposure to PFAS levels including Perfluorooctanoic acid (PFOA) and perfluorooctane sulfonic acid (PFOS), impairs insulin signaling and glucose homeostasis thereby causing maternal health issues like GDM [61, 62]. For example, female CD-1 mice exposed to low doses of PFOA *in utero* had higher levels of leptin and insulin in their serum at 21 to 33 weeks [63]. This was also true in humans where women with GDM who had a family history of T2DM revealed a substantial positive relationship with PFAS congeners Perfluoroheptanoic acid (PFHpA), Perfluorododecanoic acid (PFDoDA), Perfluorononanoic acid (PFNA), and PFOA [64]. Similarly, another study showed that both impaired glucose tolerance and GDM were positively correlated with plasma PFOS concentrations [65]. A study of pregnant Danish women showed a significant association between impaired glycemic index and elevated serum Perfluorohexane sulfonic acids (PFHxS) and PFNA concentrations [66]. PFAS exposure during pregnancy has also been linked to aberrant GWG as noted by two different human studies [67, 68], and excessive GWG can increase the risk of GDM. PFAS has been shown to interact with PPARα, γ, and β/δ [69] to induce adipogenesis, leading to obesity among pregnant women, which further impairs glucolipid metabolism [70] (Fig. 3). PFAS also acts via other PPAR-independent mechanisms to cause metabolic alterations like glycogen depletion and mitochondrial dysfunction, which was seen in pregnant mice exposed to PFAS [71]. Apart from the well-established role of the thyroid gland in regulating glucose metabolism, cardiovascular function, and storage and expenditure of energy [72], it can also influence pregnancy outcomes and fetal development. Importantly, as reviewed in an article by Birru et al., PFAS exposure in pregnant women can target the hypothalamic-pituitary-thyroid axis and this can contribute to GDM development [13] (Fig. 3). Specifically, several human studies have found that maternal PFAS levels were positively associated with thyroid stimulating hormone (TSH), thereby disrupting thyroid homeostasis, which can alter downstream glucose metabolism [73–75].

PFAS exposure can be transferred from mother to child via the placenta and breast milk [76, 77]. In fact, Kupsco et al., analyzed the ExomiRs in breast milk from mothers exposed to PFAS and found that PFOS and PFNA were associated with variable expression of certain groups of miRNAs [78]. There is more extensive literature on the association of human PFAS exposure with DNA methylation changes in either maternal serum or umbilical cord blood samples both at the global [79] as well as candidate gene levels [80, 81] (Table 2). As reviewed previously, prenatal exposure to certain PFAS is associated with adverse effects on both the mother and the infant, such as HDP, including preeclampsia, and low birth weight [82]. This PFAS exposure of the offspring can also disrupt thyroid function and cause kidney disease, obesity, metabolic and reproductive disorders [82].

### Heavy metals

In 2012, EDCs were divided into several categories by the World Health Organization (WHO). Of these categories, the detrimental impact of four heavy metals and their conjugates, namely cadmium (Cd), arsenic (As), lead (Pb), and manganese (Mn), have received the most attention [83]. The sources of these heavy metal contaminations include fertilizers, food packaging, industrial wastes, and fossil fuel combustion [83]. Biomagnification of metals as a result of dumping of industrial wastes into water bodies without proper sewage treatment by these industries serves as a major source of heavy metal exposure [83]. When As and Cd enter the body of an individual, they primarily accumulate in the kidneys, liver, and pancreas, where they affect the specific function of important enzymes and have a negative impact on glucose metabolism, including glycolysis, glycogenesis, and gluconeogenesis [84]. Various human studies have shown that exposure to heavy metals, either alone or in combination, increases the risk of developing diabetes [84, 85].

A study by NHANES demonstrated a positive correlation of human urinary Cd levels with both BMI and waist circumference in children and adolescents [86]. More specifically, women with a higher concentration of pre-pregnancy urinary Cd values were more susceptible to developing GDM [87]. While the direct evidence for the effects of Cd on obesity is not entirely clear from animal studies, there is a defined role of Cd in impairing adipose tissue physiology in vivo [88]. In vitro, Cd exposure significantly decreased the cell viability of 3T3L1 cells in a dose-dependent manner. Cd exposure was also linked with adipose cell dysfunction including reduced fatty acid synthesis and lipid degradation [89]. This occurs due to dysregulated transcription factors, like PPARγ and CEBPα [90], which cause endocrine dysfunction and alter adipocyte glucose handling, resulting in insulin resistance [88].

In 2020, Salmeri et al. conducted a systematic review and meta-analysis on maternal As exposure and GDM and reported that there is a positive correlation between both maternal blood and urine As levels with GDM [91]. Mechanistically, Yang et al. demonstrated that exposure of pancreatic-β cells to inorganic arsenite (iAs3+) activates *Nrf2*, which reduces reactive oxygen species (ROS) signaling triggered by glucose, inhibits glucose-stimulated insulin production and impairs pancreatic β-cell function [92]. Furthermore, 8-week male C57BL/6J mice, exposed to arsenite had an impairment in glucose metabolism due to miR-191 induced decrease in GLUT4 translocation and inhibition of IRS1/AKT pathway [93]. Similarly, a French cohort study evaluated how GDM in women without any previous history of diabetes was affected by prenatal exposure to Pb and Cd and found that exposure to these heavy metals was positively associated with GDM [94]. Exposure to some heavy metals, such as As and Cd, may exacerbate the “diabetogenic environment of pregnancy.” [95] These metals were positively correlated with GDM prevalence and identified in meconium samples from newborns of women with GDM [95]. Heavy metal exposure alters several other factors contributing to GDM including oxidative stress, inflammation, PPARγ suppression, and alteration of genes associated with diabetes [96].(Fig. 3).

There is also a link between prenatal heavy metal exposure and epigenetic alterations in both the placenta and cord blood. For example, As exposure in early pregnancy showed a strong correlation with DNA methylation in newborn’s cord blood DNA [97]. Similarly, early pregnancy Cd exposure led to DNA methylation alterations at specific regions and these changes in offsprings were sex- as well as race-dependent [98]. Lastly, Pb exposure prenatally caused DNA hypomethylation in humans [99] and also caused alterations in miRNA expression in animal models [100]. By altering the epigenetic mechanisms, early exposure to these heavy metals can result in detrimental long-term consequences including diabetes, obesity, and other diseases among childern [101].

### Dioxins

Dioxins are a category of organic compounds that constitute organoleptically undetected derivatives of oxanthrene as well as fumarates [102]. Dioxins generally include Polychlorinated dibenzo-dioxins (PCDD), Polychlorinated dibenzofurans (PCDF), and 2,3,7,8-tetrachloro-dibenzo-p-dioxins (TCDD). They are discharged into the atmosphere during burning of hazardous occupational wastes in open pits [103].. A part of the dioxins are metabolized and eliminated from the human body while the rest gets stored in adipose tissue as body fat [102]. Hepatic P4501A1 enzyme oxygenates lyophilic compounds like dioxins by binding with ARNT, a xenobiotic-responsive element. It then gets translocated to the nucleus and increases the expression of *Cyp1a1* gene [104], which is responsible for converting polyacrylic aromatic hydrocarbons and aromatic amines into reactive metabolites. Dioxins are primarily classified as carcinogenic but they have also been known to cause non-carcinogenic complications like atherosclerosis, HDP, and GDM [104].

A multi-center prospective study evaluated the association of organic pollutants, including dioxins, and GDM risk, in over 2000 women during early pregnancy and found a positive association between chlorinated polychlorinated biphenyls (PCBs) and GDM [64]. Similarly, another study found that women with high serum levels of PCBs had higher odds of developing GDM [105]. Dioxins have a similar structure to steroid hormones and can mimic natural hormone function, affecting the hypothalamus-pituitary axis and altering the endocrine functioning [102]. Recent shreds of evidence suggest that aryl hydrocarbon receptor (AHR) plays a vital role in the pathomechanism of dioxin intoxication [102]. Dioxins like TCDD block the synthesis of AHR and modulate the expression of estrogen-dependent genes leading to delayed embryonic development [102]. Experiments in genetic models of *Ahr* null mice demonstrate that deletion of *Ahr* leads to a significant reduction in plasma insulin as well as an impairment in insulin sensitivity [106]. Furthermore, when pregnant mice were exposed to TCDD, they showed significant downregulation of serum adiponectin levels, thereby leading to an increase in GWG [107].

Blood levels of dioxin-like PCBs significantly correlate with the expression of miR-191 in pregnant women residing in areas with high environmental levels of dioxins [108] (Fig. 2). miR-191 is known to impair insulin signaling by decreasing the translocation of GLUT4 in human fetal hepatocytes. Human peripheral blood mononuclear cells (PBMCs) also demonstrate a dioxin-induced increase in miR-191. (Fig. 3). Dioxins can also alter the expression of miR-103 and miR-107 in primary human lung fibroblasts [103] (Fig. 2). The overexpression of miR-103 and miR-107 directly regulates insulin sensitivity and obesity by augmenting the expression of enzymes such as G6Pase, PEPCK, PC, and FB-(1,6)-Pases (Table 2). These enzymes act as indicators of increased gluconeogenesis and are the primary cause of elevated glucose levels [109], which contributes to the progression of GDM. Apart from miRNA expression changes, prenatal exposure to dioxins like TCDD also augment global DNA methylation [110] as well as acetylated H3 levels [111]. Thus, dioxin exposure poses a risk for transgenerational effects due to epigenetic alterations that will also affect future generations [112].

### Parabens

Parabens are aliphatic esters of para-hydroxybenzoic acid which are widely used in pharmaceuticals, cosmetics, and as food preservatives to avoid the growth of hazardous bacteria and molds [113].. The most common parabens include methylparaben (MeP), ethylparaben (EtP), propylparaben (PrP), butylparaben (BuP), and benzyl-substituted para-hydroxybenzoic acid ester (BzP).

In a study, Ying et al., demonstrated that PrP concentrations were considerably higher in the urine of GDM patients with respect to normal pregnancies [114]. This is attributed to the stronger estrogenicity of PrP due to the presence of a side alkyl chain. PrP was also found to be associated with higher pre-pregnancy BMI leading to overweight/obesity among women [114]. Another study of over 1000 pregnant women found a correlation between urinary EtP levels and GDM [115], while another cohort study found a positive association between BuP and glucose levels in both first and second trimester [116]. In vitro studies show that a mixture of EDCs containing parabens had an adipogenic effect on 3T3L1 cells, murine preadipocyte cell line, defined by oil red O staining [117]. This effect was mediated by induction of transcription factors including CEBPα and PPARγ. MeP also increased glucose uptake and reduced lipolysis in 3T3L1 cells [118]. These adipogenic effects can lead to GWG, causing women to become more prone to GDM. Parabens also affect thyroid function as was shown by a study that found that the level of triiodothyronine (T3) was negatively associated with BuP level while a decreased concentration of free thyroxine (FT4) was seen among patients with higher PrP concentrations [119], which indirectly affects the development of both obesity and insulin resistance (Fig. 3).

Parabens influence pregnancy outcomes by altering the expression of maternal microRNAs like miR-15a-5p and miR-185 [120] thereby causing oxidative stress, apoptosis, and insulin growth factor dysfunction in the placenta thereby altering the expression of placental miRs. MiR-15a-5p was shown to be upregulated in the skeletal muscles of women with GDM [121]. It targets UCP2, which normally reduces mitochondrial ROS formation (Fig. 3). MiR-185 levels were demonstrated to be lower in the serum and placenta of women with GDM, suggesting that this miRNA could act as a non-invasive biomarker [122]. MiR-185 is also strongly associated with lipid metabolism and cholesterol homeostasis and thus, can influence adiposity and GWG. The levels of miR-185 also correlate with HOMA-IR and altered expression of miR-185 is directly proportional to the expression of key insulin signaling components like IRS-1, IRS-2, PI3K, and AKT2, which promotes insulin resistance [123] (Fig. 2). Beyond miRNAs, high paraben levels reduce DNA methylation at the *Igf2* locus in the human placenta [124] and also alter sperm DNA methylation in rat models [125], suggesting a role for DNA methylation (Table 2), altered by the effect of parabens, to be passed from parents to offspring. Interventions on reducing exposure to these parabens like EtP and BuP by lifestyle modifications can positively greatly influence maternal thereby decreasing the prevalence of severe pregnancy outcomes [126].

### Other relevant endocrine disruptors

Several other EDCs contribute to a pivotal role in the incidence of insulin resistance in pregnant women. Among these, some notable ones, as designated by the UNEP (United Nations Environment Programme) [83], and the effect of their exposure on pregnancy outcomes are discussed in this section.

Organo-chlorinated pesticides like p, p′-dichlorodiphenyldichloroethylene (DDE) and p, p′-dichlorodiphenyltrichloroethane (DDT) are strongly associated with GDM. The presence of these chlorinated pesticides in the serum of women was found to be associated with increased risk of GDM, and these compounds are small enough to expose the fetus [127]. Mechanistically, these pesticides have induced pre-adipocyte differentiation in 3T3L1 cells by increasing the protein expression levels of C/EBPα, PPARγ, AMPKα, and ACC, thereby leading to obesity [128].

Occupational and environmental exposure to dicarboximide fungicides like vinclozolin, act as anti-androgen substances and play a significant role in disrupting the normal intra-uterine environment, thereby elevating the risk for the onset of reproductive, metabolic, and behavioral disorders as demonstrated by a multi-generational rat model [129]. Another murine study showed that dietary exposure to fungicides like procymidone lead to adverse pregnancy outcomes like growth restriction and caused alterations in glucolipid metabolism in the F1 generation [130].

Exposure of pregnant rats to herbicides like linuron produces anti-androgenic activity and affects the placental-fetal unit, acting as a causative agent for fetal growth restriction and defects in male sexual differentiation [131]. Drinking water acts as one of the prominent modes of exposure to herbicides and women exposed to herbicidal nitrates before and during pregnancy have an increased risk for not only GDM but also other pregnancy complications like small-for-gestational-age babies [132].

Flame retardants like Poly-Brominated Diphenyl Esters (PBDE) are also endocrine disruptors that interfere with the intra-uterine environment of pregnant women. Maternal PBDE exposure was shown to increase the risk of cryptorchidism in a case-control study [133] and this was also associated with other maternal and fetal complications like GDM, low birth weight, and prematurity [133]. Evidence from a human trial demonstrates that PBDEs readily cross the placenta and interfere with fetal liver development [134].

Exposure to alkylphenol ethoxylates like p-nonylphenols can interfere with normal pregnancy outcomes, as was seen in a cohort study demonstrating women with increased levels of urinary nonylphenols had a significantly shorter gestation period [135]. The study further showed that nonylphenols significantly related to increased markers of oxidative and nitrosative stress in the placenta in pregnancies leading to HDPs like pre-eclampsia. Additionally, urinary nonylphenols levels in women during all three trimesters are inversely correlated with maternal weight gain, which leads to further pregnancy complications like small-for-gestational-age babies and preterm births [136].

## Conclusion

The widespread use of EDCs across the globe has given rise to an inflation in the occurrence of GDM. Various pieces of evidence suggest that EDCs like BPA, phthalates, PFAS, heavy metals, and others perturb signaling pathways, leading to GWG and GDM. EDC exposure during pregnancy further transmits these harmful chemicals to the fetus via the placenta, thus inducing pregnancy-related complications such as preeclampsia, preterm birth, and other metabolic, cardiovascular, and reproductive disorders among the infant. This propagates a vicious cycle by having a lasting transgenerational impact. However, there is a dearth of epigenetic and translational studies associating EDC exposure and the development of GDM among pregnant women. Importantly, to manage the rising burden of GDM, research focus needs to be shifted toward clinical actions such as using various Exo-miRs as biomarkers to aid in early screening as well as timely intervention of GDM at the community level.

The deployment of integrated technologies such as federated-based learning systems using m-health platforms will analyze big data on a spatiotemporal scale, which forms the rationale for the precision-oriented detection of GDM along with automated clinical resource allocation. It will also increase our understanding of the patterns and processes associated with the preponderance of niche-specific risk factors as well as variations in susceptibility to EDC-induced GDM. We believe that future research should focus on the detection of the EDC-specific ExomiRs for the diagnosis of GDM and identification of emergent GDM hotspots at the community level. This would provide advice to the administrators at the local, regional and national levels to develop and deploy niche-specific policies and programs. Finding the research gaps associated with EDC-induced GDM and working towards reducing them will help in significantly alleviating the burden of GDM. Furthermore, since ExomiRs act as upstream regulators of proteins [137], an estimation of dysregulation in the expression levels of the EDC-associated ExomiRs in the first trimester of pregnancy can lead to better management of GDM. As of the currently practiced approaches like the oral glucose tolerance test (IADPSG criteria: fasting plasma glucose ≥ 92 mg/dL, 1 h plasma glucose ≥ 180 mg/dL, 2 h plasma glucose ≥ 153 mg/dL) and estimation of protein biomarkers of GDM like HbA1c (5.45%) [138–141]. These various screening criteria which are presently in use in clinical practice for the diagnosis of GDM generally detects the pathological condition in patients post-20 weeks of gestation with the earliest detection being reported around 13–16 weeks of pregnancy [142]. This increases the necessity for the early diagnosis of GDM thereby leading to better management as well as reduction in the burden of GDM.

## Data Availability

Not applicable.
